# Newer Generation Immunoassays in Screening for Adrenal Insufficiency: Comparison of Baseline Morning Cortisol Levels With Cosyntropin Stimulation Testing

**DOI:** 10.1016/j.aed.2026.03.001

**Published:** 2026-03-11

**Authors:** Ekta Shrestha, Stewart G. Albert, Allina Ghimire, Shellsea Portillo Canales, Iqra Iqbal, Rukayat Akande

**Affiliations:** Department of Internal Medicine, Division of Endocrinology, SSM Health/ Saint Louis University School of Medicine, St. Louis, Missouri

**Keywords:** adrenal insufficiency, cosyntropin stimulation test, new generation cortisol assays, Addison’s disease, adrenal gland, adrenal testing

## Abstract

**Objective:**

Serum cortisol immunoassays with improved specificity for cortisol have prompted revisiting the definition of a normal response at 60-minute cosyntropin stimulation testing (CST-60) to ≥14 μg/dL. European and American Endocrine Societies define values of AM cortisol >10 μg/dL as adequate to rule out adrenal insufficiency (AI) in patients with long-term use of corticosteroids. We sought to compare AM cortisol levels with CST-60 in patients suspected of AI from various causes.

**Methods:**

A retrospective review was performed from the electronic health record of a large midwestern hospital in patients who were evaluated for AI.

**Results:**

There were 95 patients (51women, age 58.8 ± 17.8 SD years) with 43 tests performed outpatient, 48 inpatient, and 4 ICU settings. The pretest diagnoses were adrenal abnormalities (*n* = 10), hyponatremia (*n* = 11), hypoglycemia (*n* = 9), fatigue (*n* = 19), hypotension (*n* = 35), pituitary abnormality (*n* = 7), and shock (*n* = 4). Binary logistic regression analyses and receiver operating curves compared AM cortisol as predictors of a normal CST-60 ≥ 14.6 μg/dL and to the previous criteria of a CST-60 ≥18.0 μg/dL. Receiver operating curves showed that an AM cortisol of 11.1 μg/dL had a specificity of 93% for a CST-60 >14.6 g/dL. Using historical levels of CST-60 ≥ 18.0 μg/dL, there would be 17% false positives requiring unnecessary dynamic testing.

**Conclusion:**

In this community setting with low risk of AI (16%), a baseline AM cortisol of 11.1 μg/dL can reliably preclude the need for a CST-60. Clinicians should be aware of the new cutoffs for the assays available to them when evaluating patients for AI.

**Highlights**

• Serum cortisol immunoassays with improved specificity for cortisol have prompted revisiting the definition of a normal response at 60-minute cosyntropin stimulation testing (CST-60) to 14 μg/dL
• We sought to compare AM cortisol levels with CST-60 in patients suspected of adrenal insufficiency from various causes with retrospective review using newer generation immunoassays
• We found AM cortisol of 9.0 μg/dL had a positive predictive value of 95% for CST-60 14 μg/dL
• Receiver operating curves showed a range of AM cortisol of 9.4 to 11.0 μg/dL with a specificity of 93%
• Clinicians should be aware of the new cutoffs for the assays available to them when evaluating patients for adrenal insufficiency

**Clinical Relevance**

An AM cortisol of ∼9 to 11 μg/dL reliably excludes adrenal insufficiency in low-risk patients using modern assays, aligning with 60-minute cosyntropin stimulation testing ≥14 μg/dL. This reduces unnecessary cosyntropin testing driven by outdated ≥18 μg/dL thresholds, enabling faster, cost-effective, and assay-specific clinical decision making.

## Introduction

Adrenal insufficiency (AI) is a potentially life-threatening disorder that requires a timely and accurate diagnosis.^1^^,^^2^ Failure to identify AI can lead to severe clinical consequences, including adrenal crisis and death. Overdiagnosis of AI may result in unnecessary treatment with steroids with long-term complications, including suppression of the hypothalamic-pituitary-adrenal axis.^3^^,^^4^

Advancements in immunoassays have significantly improved the specificity of serum cortisol measurements.^5^, ^6^, ^7^, ^8^, ^9^ Older radioimmunoassay using polyclonal antibodies are known to detect cortisol-like compounds such as cortisone and prednisolone. The historical threshold for a normal response in polyclonal antibody assays to a cosyntropin stimulation testing (CST) was a cortisol at 60 minutes (CST-60) of ≥18.0 μg/dL. The newer monoclonal assays (eg, Elecsys II and Architect) have minimal cross-reactivity with other glucocorticoids.^5^ As a result, revised guidelines established a lower cortisol cutoff threshold of ≥14.6 μg/dL 60 minutes after CST for defining the normal range of responses and ruling out AI.^6^^,^^9^ Thus, assay-specific cutoffs would minimize misclassification and prevent overtreatment in suspected cases of AI.^4^

Further studies have been conducted to evaluate the accuracy of morning cortisol versus CST in reducing the need for dynamic testing.^10^, ^11^, ^12^, ^13^, ^14^ Thus, the 2024 combined European and American Endocrine Societies have established criteria for persons who have undergone chronic corticosteroid therapy that a morning cortisol level <5 μg/dL is considered abnormal, whereas levels above 10 μg/dL are sufficient to rule out AI and would not require further CST.^3^

Building on these screening criteria, we sought to determine whether these morning cortisol measurements may serve as valid indicators of adrenal reserve in individuals suspected of having AI from any cause who were also undergoing CST.

## Methods

We collected data using the electronic health record Epic (Slider/Dicer subprogram) from a large midwestern academic and community-based practice (Sisters of St Mary, SSM Healthcare) using time periods between January 1, 2024 and June 30, 2024. Search criteria were men and women, over 18 years of age, who had determination of morning (AM) serum cortisol levels and serum cortisol levels at 60 minutes after a CST. The charts were reviewed for the patients’ age, sex, body mass index, weight, and diagnosis as per the decisions of the health care providers. All clinical chemistry techniques and assays were reviewed to verify that the tests were performed using newer generation cortisol assays. The Abbott Alinity Cortisol assay is a chemiluminescent microparticle immunoassay used for the quantitative determination of cortisol in human serum and plasma which had been implemented since December 23, 2023 in all SSM Healthcare facilities and the normal response at 60 minutes is ≥14.6 μg/dL.

### Statistical Analysis

The primary end point of the study was to evaluate the ability of morning cortisol determinations to predict a normal response at 60 minutes after a CST. Baseline demographics, initial diagnostic indications, and location of testing (inpatient, outpatient, ICU) were compared by *t*-tests or analysis of variance with post hoc analysis by Fisher’s least significant difference. Correlation and regression analysis were performed by Pearson testing. Comparison of proportions was done by Χ^2^ test. Binary logistic regression was performed to calculate positive predictive values, and receiver operating curves (ROCs) were generated to derive sensitivity and specificity. All procedures were performed on SPSS v 29.0.0.0. Significance was defined as *P* < .05 by two-tailed testing. The study data was considered exempt for human research by the St Louis University Institutional Review Board as the data had been anonymized.

## Results

A convenience sample was retrieved of 100 patients who had CSTs. Five patients were excluded because they only had cortisol levels determined in the evening. Of the 95 patients, there were 44 men and 51 women, with a mean age 58.8 (±17.8 SD) years. Men were older than women (64.2 ± 14.2 years vs 54.2 ± 19.3 years, *P* = .01). The tests were performed as outpatient (*n* = 43), inpatient (*n* = 48), and ICU (*n* = 4) settings. The pretest diagnoses were presumed adrenal abnormality (*n* = 10): congenital adrenal hyperplasia, adrenal surgery, adrenal abnormality on imaging); hyponatremia (*n* = 11); hypoglycemia (*n* = 9); fatigue (*n* = 19); hypotension (*n* = 35); pituitary abnormality (*n* = 7) and shock (n = 4).

**Fig. 1:**
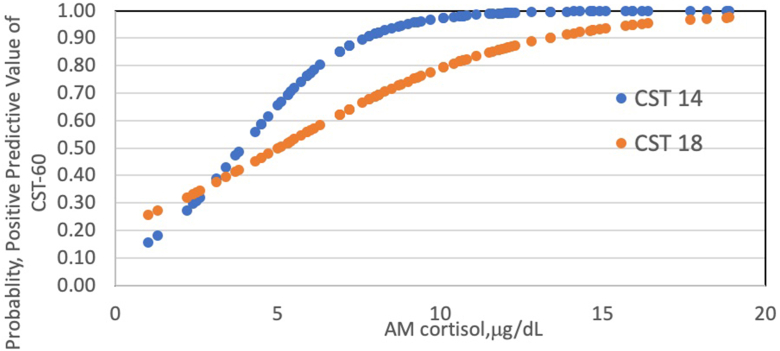
Binary logistic regression of positive predictive value of morning cortisol vs a normal response in a subsequent 60-minute value of cosyntropin stimulation test (CST-60). Red line is the probability of a normal CST using newer cut of values for CST-60 cortisol levels of≥14.6 μg/dL, and blue line is the probability of a normal CST-60 using a historical cutoff ≥18 μg/dL. Dashed line is shown as the mean AM cortisol in this convenience sample.

The mean AM cortisol from the convenience sample was 10.2 ± 5.5 μg/dL. A binary logistic analysis curve was then used to calculate the probability that this sample would be sufficient for power analysis (Fig. 1). There was a 97% probability that a morning cortisol of ≥ 10.2 μg/dL would predict a normal response at CST-60 of cortisol ≥14.6 mg/dL. Using the older cutoff from a CST-60 of cortisol ≥18.0 μg/dL, an AM cortisol value of ≥10.2 μg/dL would have an 80% probability of having a normal response.

**Fig. 2:**
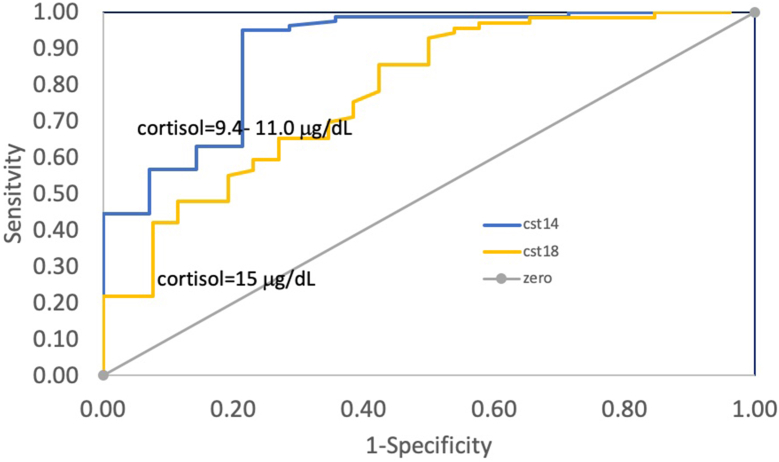
Receiver operating curves (ROC) of probability (positive predictive value, PPV) of a normal 60-minute value of cosyntropin stimulation test (CST-60) for an AM cortisol vs CST-60. (*A*) ROC line is a comparison using the newer cortisol cut point value of ≥14.6 μg/dL. (*B*) ROC line using the historical cortisol cut point of ≥18.0 μg/dL. Diagonal red line is line of identity. Vertical lines show 95% specificity, 5% false positive rate.

To calculate the sensitivity and specificity of the morning cortisol levels to predict an adequate response to the CST-60, a further analysis was performed using ROCs. For the newer guidelines of a CST-60 of ≥14.6 μg/dL, a morning cortisol of 11.1 μg/dL would have a sensitivity of 44% and a specificity of 93% to rule out AI (Fig. 2 *A*). Using the older guidelines of post CST-60 cortisol of ≥18.0 μg/dL, a morning cortisol of 15.0 μg/dL would have a sensitivity of 22% and a specificity of 96% to rule out AI (Fig. 2 *B*). There was one false negative test (rate = 1%) with an AM cortisol 11.1 and lack of response to CST-60 with a 60-minute cortisol = 13.6 μg/dL.

The presumed diagnosis of AI was defined as an abnormal CST-60 with cortisol <14.6 μg/dL was 15/95 = 15.8%, whereas using the older criteria of CST-60 <18.0 μg/dL was 26/95 = 27%.

**Fig. 3:**
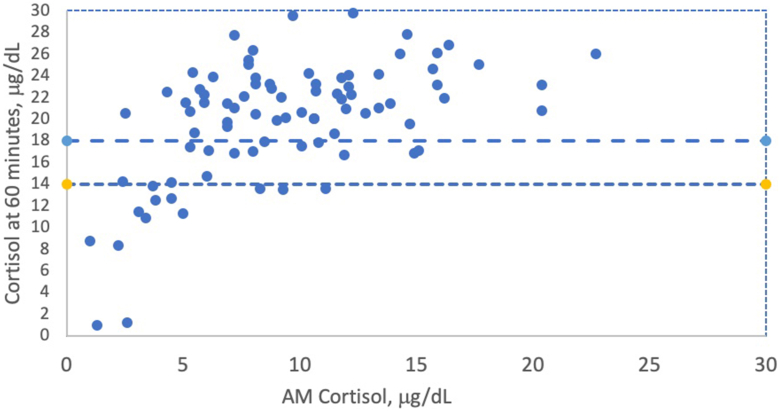
Comparison of AM cortisol with subsequent 60-minute value of cosyntropin stimulation test (CST-60). Points between new cutoff values of CST-60 of ≥14.6 μg/dL and historical values of ≥18.0 μg/dL are false positive tests that would have required dynamic testing.

These comparisons of morning cortisol levels and CST-60 results are shown in Fig. 3. If using the older guidelines, there would be 11 false positives (12%) which would require further dynamic testing. In this sample of community patients who were at low risk of AI (15.8%), a baseline morning cortisol within the range of 11.1 μg/dL precluded the need for a CST to predict a normal CST-60 cortisol of ≥14.6 μg/dL.

There were 21 patients who received chronic steroid use for >3 months who were included in the analysis. There were no differences in this subgroup compared to the nonsteroid users with regard to age, morning cortisol, or adrenocorticotropic hormone levels. They had lower CST-60 responses (21.0 ± 7.0 μg/dL vs 23.2 ± 7.5 μg/dL, *P* < .001). A subnormal response to CST-60 <98 μg/dL was found in 7 of the 21 on chronic steroids (33%) compared to 7 of 74 (9.5%) without steroids (*P* = .012). Similarly 11 of those on steroids (52%) compared to 15 of those without steroids (20.3%) had subnormal CST-60 with a cutoff of 18 μg/dL (*P* = .005).

## Discussion

AI can result from a primary adrenal disorder, secondary to deficiency of adrenocorticotropic hormone, or suppression of this hormone by external glucocorticoid or opioid medications.^15^ Because timely identification is critically important, there is a need for reliable, quick, and accessible diagnostic tools. Currently, early morning serum cortisol levels and dynamic testing by a CST are commonly used to evaluate adrenal function, and there have been studies which have compared AM cortisol levels with CST to reduce reliance on CST.^11^, ^12^, ^13^, ^14^

The European and American Endocrine Societies jointly have established criteria for persons who have undergone chronic corticosteroid therapy suggesting that a morning cortisol greater than 10 μg/dL is sufficient to rule out AI and hence would not require further CST.^3^ The present study questioned whether similar criteria may be used in other cases of presumed AI.

Our study demonstrated that an AM cortisol level greater than 11.1 μg/dL had >95% specificity to predict a normal CST-60 response using the newer criteria following CST. This suggests that in many clinical situations, an AM cortisol level above this threshold may be sufficient to exclude AI, potentially reducing the need for further dynamic testing.

Our findings support the 2024 Endocrine Societies guidelines, which recommend that an AM cortisol level >10 μg/dL is sufficient to confirm recovery of the hypothalamic-pituitary-adrenal axis. However, these guidelines were particularly focused on individuals who were on long-term glucocorticoids therapy. In contrast, our study includes a broader population with diverse etiologies of AI. This included congenital adrenal hyperplasia, adrenal surgery, pituitary disorders, and critical illness presentations such as shock, hypotension, and unexplained hypoglycemia. Our evaluation with morning cortisol testing using newer, more specific immunoassays should be a valuable tool in assessing AI across a broader clinical spectrum, potentially reducing the need for dynamic testing.

A limitation of our study is that it is a retrospective analysis of clinical data from a sample of community-based patients with a low risk of subnormal CST (16%). Our study results may not apply to patients with a higher risk of AI.

## Conclusion

Morning serum cortisol measured using new-generation assays is a valuable tool for ruling out AI when levels exceed 11.1 μg/dL in a population with a low pretest risk for AI. This use of a morning cortisol threshold reduces the need for dynamic testing, not only in patients with a history of long-term steroid use, but also extends the role across a broad spectrum of other potential causes of AI.^16^ Using the AM cortisol values may preclude the need for CST, which involves the cost of the CST itself (which may be estimated at $250, but that does not include the facility charges) and eliminates the time and inconvenience required by the patient. Clinicians should be aware of the new cutoffs for the assays available to them when evaluating patients for AI.

## Disclosure

The authors have no conflicts of interest to disclose.
